# Difference in Virulence of *Mycobacterium avium* Isolates Sharing Indistinguishable DNA Fingerprint Determined in Murine Model of Lung Infection

**DOI:** 10.1371/journal.pone.0021673

**Published:** 2011-06-28

**Authors:** Eduardo Pinheiro Amaral, Thereza Liberman Kipnis, Eulógio Carlos Queiróz de Carvalho, Wilmar Dias da Silva, Sylvia Cardoso Leão, Elena B. Lasunskaia

**Affiliations:** 1 Laboratory of Biology of Recognition, Universidade Estadual do Norte Fluminense, Campos, Rio de Janeiro, Brazil; 2 Laboratory of Animal Morphology and Pathology, Universidade Estadual do Norte Fluminense, Campos, Rio de Janeiro, Brazil; 3 Laboratory of Immunochemistry, Instituto Butantan, São Paulo, Brazil; 4 Department of Microbiology, Immunology and Parasitology, Universidade Federal de São Paulo, São Paulo, Brazil; Tulane University, United States of America

## Abstract

**Background:**

Opportunistic *Mycobacterium avium* typically causes disease in immunocompromised patients and in some groups of apparently healthy individuals. The high virulence of some bacterial lineages increases the disease risk. High-resolution molecular genotyping studies of *M. avium* clinical isolates demonstrated that some genotype patterns were more prevalent than others, suggesting that close genetic relatedness of these successful isolates sharing a similar genotype could determine similar biological properties associated with high virulence.

**Methods and Findings:**

In this study, we aimed to compare the virulence and pathogenic properties of two epidemiologically unrelated *M. avium* isolates sharing an indistinguishable DNA fingerprint in a well-characterized model of pulmonary infection in mice, resistant or susceptible to mycobacteria. The mice, C57BL/6 wild- type or IFN-gamma gene disrupted (GKO), respectively, were intratracheally infected with two isolates, H27 (human blood isolate) and P104 (pig lymph node isolate), and the lungs were examined for bacterial loads, histopathology and cytokine gene expression. The obtained data demonstrated significant differences in the virulence properties of these strains. Although the H27 strain grew significantly faster than P104 in the early stage of infection, this bacterium induced protective immunity that started to reduce bacterial numbers in the wild- type mice, whereas the P104 strain established a chronic infection. In the GKO mice, both strains were capable of causing a chronic infection, associated with higher bacterial burdens and severe lung pathology, in a similar manner.

**Conclusions/Significance:**

The results demonstrated that the studied isolates differed in the pathogenic properties although were indistinguishable by actually widely used genotyping techniques demonstrating that the genotype similarity does not predict similarity in virulence of *M. avium* isolates.

## Introduction

Infection with opportunistic *Mycobacterium avium* subsp. *hominissuis* is a significant health problem among HIV-infected populations [1], children with genetic deficiencies in IFN-γ and IL-12 production or function [2], in individuals with a prior history of lung pathology and among subsets of apparently healthy people, such as thin elderly women [3], [4]. Pulmonary disease is a most common manifestation of *M. avium* infection in immunocompetent individuals [5], whereas AIDS patients frequently present a generalized infection [6]. Increased susceptibility of the determined populations to *M. avium*, as well as enhanced virulence of some bacterial strains, increases the probability of disease development. The precise modes of transmission of *M. avium*, as well as the main sources of human infection, are not well elucidated.

The high-resolution pathogen genotyping, based on restriction fragment length polymorphism (RFLP) with IS*1245* and IS*901*, is a standardized technique of the *M. avium* genotyping that was widely employed for the epidemiological purposes over the past twenty years [7]. This method was used for the identification of *M. avium* isolates from different sources demonstrating significant differences between isolates obtained from birds and mammals, including humans and pigs [8], [9]. Bird isolates were characterized by typically low IS*1245* copy number patterns and by the presence of IS*901*, whereas isolates obtained from humans and pigs presented polymorphic multiband IS*1245* genotype patterns and the absence of hybridization with the IS*901*-specific probe [8]. These and others genotype particularities, as well as different growth-temperature tolerance characteristics, were used as a base to suggest the separation of two evolutionarily conserved taxa of *M. avium,* designated *M. avium* subsp. *avium* for bird-type isolates and *M. avium* subsp. *hominissuis* for the human/porcine type of *M. avium* [10]. Further analysis of genotype characteristics associated with pathogenic isolates of *M. avium* demonstrated that some genotype patterns were more prevalent than others. Pathogenic *M. avium* isolates genotypically identical to the genome sequence strain P104 were identified by a two-step approach based on large sequence polymorphism (LSP)-based genotyping test associated with high-resolution repetitive sequence-based PCR typing. These isolates were obtained from ten different patients at five clinical sites in the western United States of America over a 17-year time span [11], suggesting that some strains can be isolated from single or multiple environmental sources over extended time periods or those patients may become asymptomatically colonized by *M. avium* pathogenic strains. In another study performed in Brazil, a large cluster of *M. avium* isolates obtained from pigs and human patients, exhibiting indistinguishable DNA fingerprinting pattern as determined by IS*1245*-based RFLP and PCR-restriction enzyme analysis of *hsp65* (PRA-*hsp65*) methods, was identified as a result of genotyping of 108 individual isolates [12]. These data suggest that the close genetic relatedness of these successful isolates sharing a similar genotype could determine similar biological properties associated with increased virulence of these strains, but this issue has never been tested in direct experiments.

Accurate evaluation of the virulence phenotype of different animal and environmental isolates of *M. avium* needs appropriate animal models. The C57BL/6 mice, wild type (WT) and selectively IFN-γ gene-deficient (GKO), were widely employed to characterize bacterial virulence after respiratory inoculation with different mycobacterial species, including clinical isolates and reference strains of *M. avium* [13], [14], and a higher susceptibility of the GKO mice to mycobacteria in comparison with the WT mice [13], [15], [16] was confirmed.

In this study, we employed the model of *M. avium* pulmonary infection in C57BL/6 mice, WT and GKO, to compare bacterial virulence and pathogenicity of two epidemiologically unrelated *M. avium* isolates, namely H27 (isolate from human blood) and P104 (isolate from pig lymph node), that in a previous study [12] were demonstrated to exhibit indistinguishable genotype patterns as determined by two widely used for the identification of *M. avium* subsp. *hominissuis* methods: IS*1245*-based RFLP and PRA-*hsp65* techniques. The obtained data demonstrated significant differences in the virulence and pathogenic properties of these two genetically closely-related isolates, suggesting that although these bacteria are most likely a direct progeny of the same bacterial strain, these isolates belong to two distinct clonal variants which could not be differentiated by genotyping tools based on detection of IS elements and single gene variation.

## Results

### Growth of two *M. avium* isolates in 7H9 Middlebrook broth and in bone marrow-derived macrophages obtained from C57BL/6 mice

**Figure 1:**
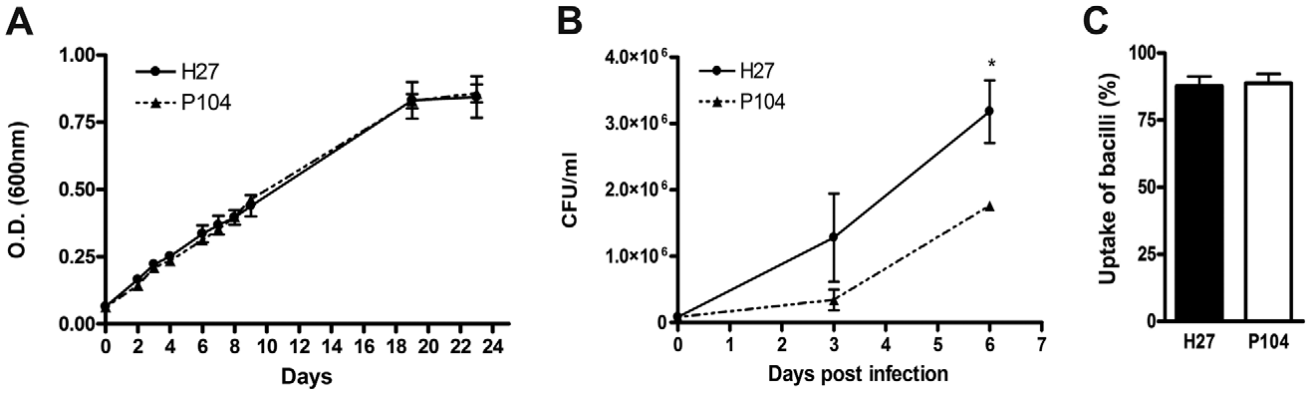
Capacity of H27 and P104 *M. avium* strains to grow in macrophages and 7H9 broth. Bacterial growth in the broth within 24 days was evaluated by spectrophotometry (A). Bone marrow cells were obtained from C57BL/6 mice and differentiated to macrophages *in vitro*. The resulted cells were infected at a MOI 1∶1 bacteria/macrophage, incubated for 3 h at 37°C, washed, tested for the mycobacterial uptake, and cultivated for additional 6 days. The resulted cells were lysed and CFU numbers (B) were quantified as indicated in Material and methods. The percentage of macrophages with internalized mycobacteria in the culture was quantified 3 h after infection by Ziehl-Neelsen method (C). The data obtained in three independent experiments are presented as mean ± SD of samples in triplicate. Asterisk represent statistical significance (*p<0.05).

In order to evaluate virulence-associated properties of two pathogenic *M. avium* isolates, H27 and P104, with previously determined genotypes, we first studied the intrinsic capacity of these bacteria to grow in the specific mycobacterial medium, 7H9 Middlebrook broth, as well as measured the bacterial capacity to intracellular growth in murine macrophages. The growth curves of H27 and P104 isolates in the broth were similar (Fig. 1A). Bone marrow-derived macrophage cultures were infected by each bacterial strain at a MOI of infection 1∶1, and the bacterial uptake was evaluated by quantification of the number of macrophages with internalized mycobacteria in the infected cultures. The obtained data demonstrated that phagocyte index in the cultures infected with H27 and P104 strains were similar (Fig. 1C). The intracellular growth rate of these strains was compared within 6 d after infection. The data demonstrated that growth of H27 in macrophages was significantly faster than that of the P104 strain (p<0.05, Fig. 1B). The number of H27 and P104 bacteria recovered from macrophages on day 0 was similar (8.0 ± 2.8×10^4^ and 8.3 ± 1.7×10^4^ CFUs, respectively), whereas by day 6 the number of H27 bacteria was at least two times higher than that of the P104 bacteria.

### Bacterial loads in the lungs, livers and spleens of C57BL/6 and GKO mice after intratracheal challenge with *M. avium* strains

**Figure 2:**
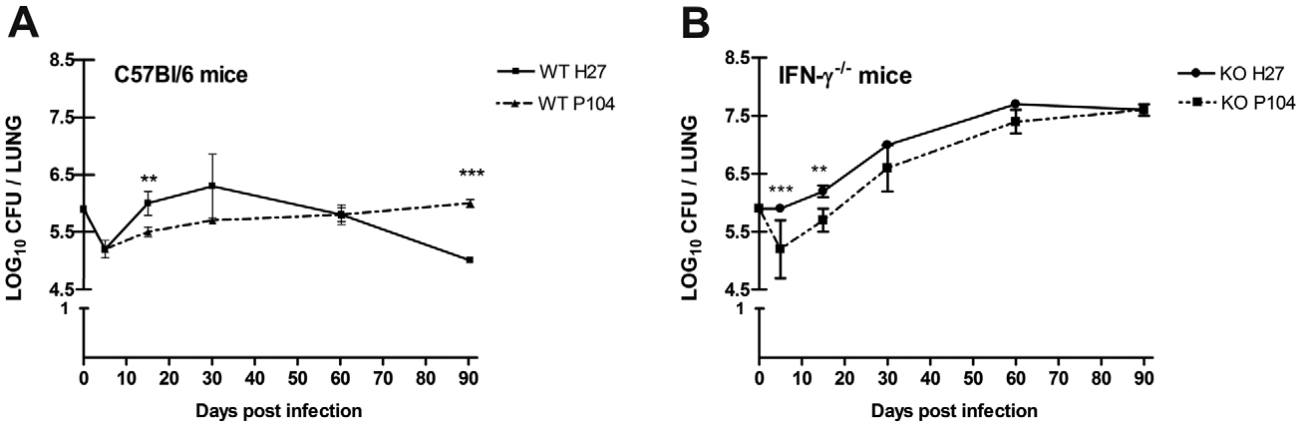
Lung bacillary loads in C57BL/6 WT and GKO mice infected with *M. avium* strains. Mice were infected by an i.t. injection of two *M. avium* strains, H27 and P104, isolated from different hosts (2.5×10^6^ bacilli). At the indicated time-points, six mice were sacrificed, and bacterial burdens (CFU) in the lungs were measured. The data were obtained in three independent experiments. Asterisks represent statistical significance (**p<0.01, ***p<0.001).

Mice were infected i.t. at a dose of 2.5×10^6^ bacilli, and the lungs, livers and spleens were examined for the number of viable bacteria. Significant differences in growth characteristics of P104 and H27 strains were observed. At the initial stage of infection, after slight reduction in the number of the both bacteria in the lungs of WT mice soon on day 5 (Fig. 2A), the growth rate of the H27 strain was higher than that of P104 strain, especially in the GKO mice (Fig. 2B). Following this, the growth of H27 strain was halted and started to drop 30 days after infection leading to significant CFU reduction (p<0.01) at 1 log within 90 days after infection (Fig. 2A) and demonstrating a declining trend in the growth of this strain. Meanwhile, bacterial numbers of the P104 strain recovered from the lungs of WT mice were permanent demonstrating that these bacteria caused sustained infection that was controlled, but not cleared, by the immune system (Fig. 2A).

The growth rate of the both strains in the GKO mice showed increase in the CFU number in all course of the infection, starting 6.0 log on day 0 and reaching in a similar manner nearly 7.5 log CFU by day 90 (Fig. 2B). Bacterial loads of the both strains in the GKO mice were significantly higher than those in the WT mice starting from day 15 after infection.

**Figure 3:**
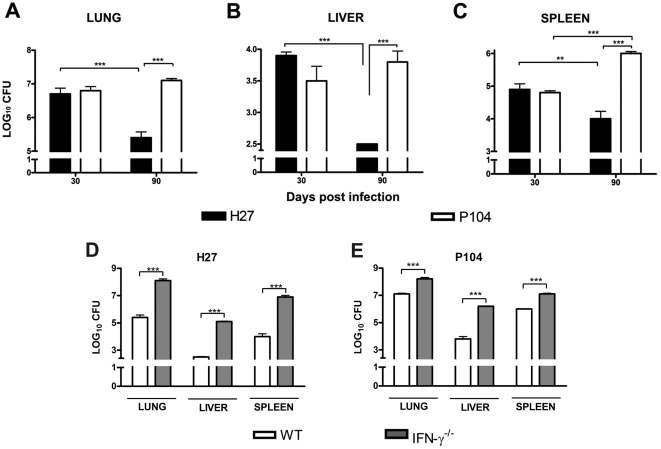
Comparison of lung, liver and spleen bacillary loads in C57BL/6 WT and GKO mice infected with *M. avium* strains. Mice were infected as indicated in the legend to Fig. 2. At the indicated time-points, six mice were sacrificed, and bacterial burdens (CFU) in the lungs, livers and spleens were measured. The data were obtained in two independent experiments. Asterisks represent statistical significance (**p<0.01, ***p<0.001).

Additionally, capacity of the immune system to clean the bacteria in three target organs, lungs, livers and spleens, was compared (Fig. 3). In the WT mice, the bacterial loads were measured on days 30 and 90 following infection. Significant reduction of the number of H27 bacteria was observed within this period in all of the tested organs (Fig. 3A, 3B and 3C), and was more pronounced in the liver were the bacterial drop was nearly by 1.5 log, in the lungs – 1.3 log, whereas in the spleen it was 0.9 log. The P104 strain demonstrated capacity to persist in all of the tested organs of the WT mice, without reduction of the bacterial number in the lung and liver and significantly increasing in the spleen.

In the GKO mice, bacterial loads in the spleens and livers were measured only on day 90 after infection and compared with those in the WT animals (Fig. 3D and 3E). In all the tested organs the bacterial numbers were higher in the GKO mice, than those in the WT mice, by 1–2 log. In the lungs and spleens of GKO mice, the number of H27 and P104 bacteria was similar, whereas it was significantly higher in the livers infected with the P104 strain (p<0.001).

### Histopathology of the lungs, spleens and livers of C57BL/6 and GKO mice after intratracheal challenge with *M. avium* strains

**Figure 4:**
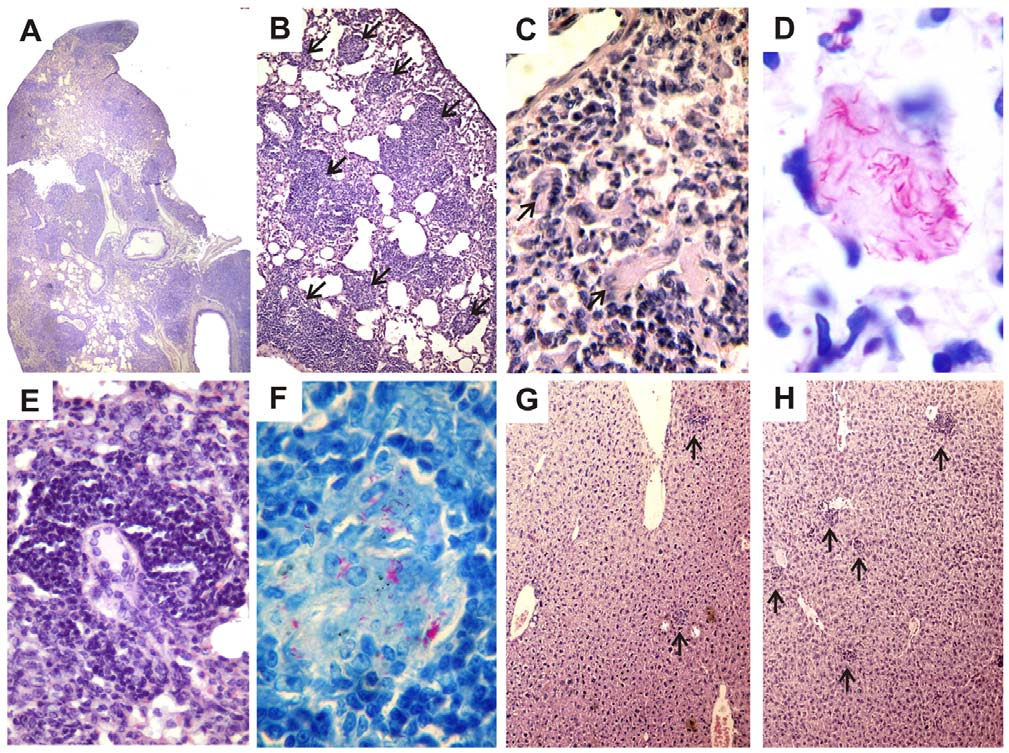
Lung, liver and spleen histopathology of C57BL/6 WT and GKO mice infected with the P104 or H27 *M. avium* strains. Representative lung histopathology at 60 days after P104 strain challenge in the WT (B) and GKO (A, C–E) mice and spleen (F) of the GKO mice. Lung presented parenchyma consolidation by epithelioid granulomatous infiltration (A), multifocal lesions with typical granuloma (B - arrows), cellular infiltration containing multinucleated giant cells (C - arrows) with intracellular fuchsinophilic acid-fast bacilli (D); granuloma containing multinucleated giant cells (E) and presence of BAAR inside the intrafollicular granuloma in spleen (F). Representative liver histopathology at 90 days after the challenge of the WT mice with H27 (G) and P104 (H) strains. The tissue samples were stained with H&E (A, B, C, E, G and H) or by Ziehl-Neelsen method (D and F). Total original magnification: A (4x); B, G, H (10x); C, E (20x); F (40x); D (100x).

Histological samples of lungs, spleen, and liver were examined at days 30, 60 and 90 following infection. The characteristics of cell infiltration and the presence of BAAR were evaluated. There was no obvious difference among histopathological changes observed in lungs and spleens of mice infected with P104 and H27 strains, therefore, the data obtained in these organs on day 60 after the challenge with P104 strain were presented as representative (Fig. 4). At day 30 after the challenge, the predominant pathologic finding in both mice strains was characterized by peribronchiolar inflammatory infiltrates of epithelioid macrophages and lymphocytes that were better organized in the granulomas of WT mice than of GKO mice (data not shown). At day 60, the differences between the lesions observed in WT and GKO were more striking. In the WT mice, numerous multifocal granulomas (Fig. 4B, arrows) were densely organized and surrounded by clusters of lymphocytes and a few foamy cells. Cellular infiltration of lungs in the GKO mice was even more robust, but few organized granulomas were observed (Fig. 4A–E). Large areas of diffuse lung consolidation were apparent with a large amount of multinucleated giant cells (Fig. 4C, arrows) and foamy cells containing abundant mycobacteria (Fig. 4D). Involvement of the spleen and liver in the infectious process was more pronounced in GKO mice than in WT mice. The former presented pronounced splenomegaly and numerous, macroscopically observed white nodules, whereas the spleen and liver in WT mice presented few macroscopically visible alterations. However, microscopically, hyperplasia of lymphoid follicles and large numbers of BAAR+ intrafollicular granulomas in the spleen (Fig. 4F) and numerous incipient granulomas in the liver (Fig. 4G–H) were observed. It should be noted that number of the granulomas in the liver of WT mice infected with the P104 strain was higher (Fig. 4H) than that of H27 strain (Fig. 4G).

In spite of the severe pathological changes observed in the WT and, especially, the GKO mice, both animal groups maintained viability up to 140 d of observation. An increase of the infection dose to 10^8^ bacilli led to the death of 60% of animals in the GKO group infected with the P104 strain within 140 d, whereas all the WT mice were still alive (data not shown).

### Expression of cytokines in the lungs of C57BL/6 and GKO mice after intratracheal challenge with *M. avium* strains

**Figure 5:**
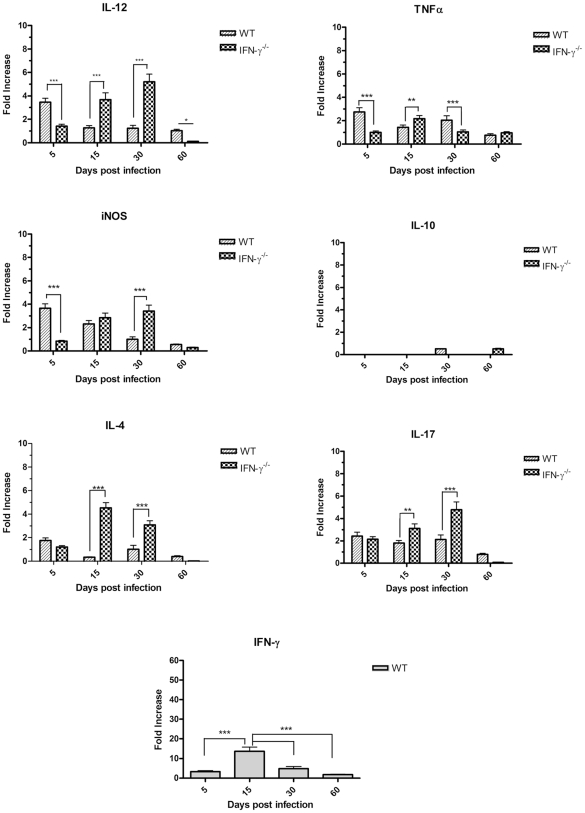
Expression of cytokine genes in lung cells of mice infected with P104 strain. C57BL/6 and GKO mice were infected as indicated in the legend to Fig.2. mRNA was extracted from lungs of these mice on days 5, 15, 30 and 60 post-infection. Levels of mRNA were quantified by real-time PCR using specific primers for inflammatory cytokine genes and normalized to the level of β-actin expression. Cytokine gene expression determination was performed with four animals per time-point. The data were obtained in two independent experiments. Results are expressed as fold increase ± SEM compared to non-infected animals. Asterisks represent statistical significance (**p<0.01, ***p<0.001) when compared WT and GKO mice.

**Figure 6:**
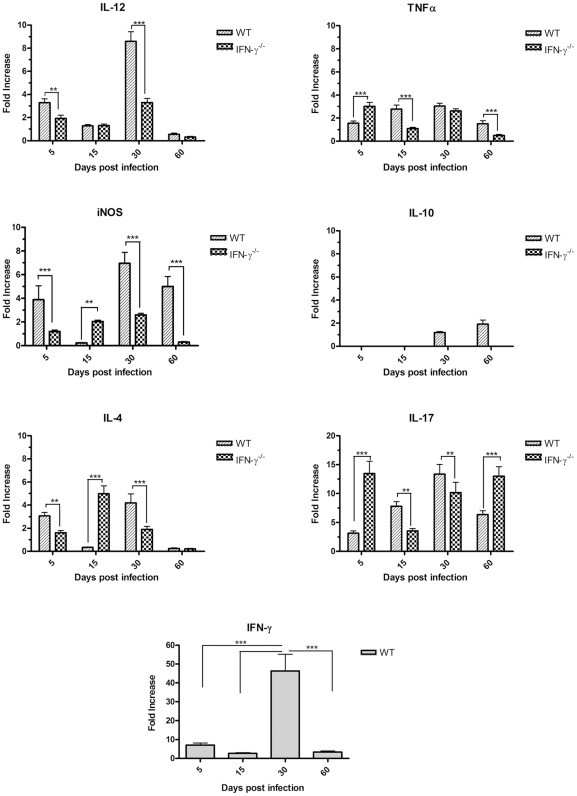
Expression of cytokine genes in lung cells of mice infected with H27 strain. Experiments were performed and results of cytokine gene expression determination were presented exactly as described in the legend to Fig. 5.

**Figure 7:**
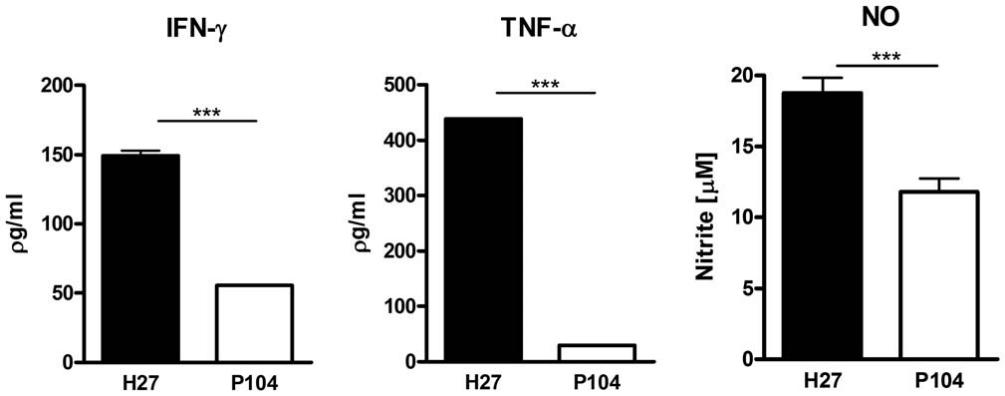
Production of pro-inflammatory mediators in culture of cells obtained from lungs of C57BL/6 WT mice infected with *M. avium* H27 or P104 strains. Lung cells were isolated on day 30 after i/t infection of mice and cultured in 96 well-plates, 5×10^4^ cells/well. Culture supernatants were collected after 48 h incubation and concentrations of IFN-γ and TNF-α were measured by sandwich ELISA. Nitric oxide production was measured by Griess reaction. The data were obtained in two independent experiments. Asterisks represent statistical significance (***p<0.001).

To characterize the immune response of the host to the studied *M. avium* strains, expression of the anti-mycobacterial defense cytokine genes was quantified in the lungs of the infected mice by real-time RT-PCR ( e first 5 d after infection, both strains had increased expression of IFN-γ, IL-4 and IL-17 in WT mice this was accompanied by macrophage activation and enhanced expression of the pro-inflammatory mediators IL-12, TNF-α and iNOS. Further into the experiment, the kinetics of cytokine expression induced by the two strains was quite different. The strain P104 was able to rapidly increase IFN-γ expression in the lungs which peaked on day 15 post-infection and further was reduced (Fig. 5). In contrast to the P104 strain, the H27 bacteria induced strong IFN-γ expression 30 d after the infection (Fig. 6) and the level of induction was three times higher than that induced by P104 bacteria on day 15. It is of importance, that the moderate increase of IFN-γ expression induced by the P104 bacteria did not enhance the expression of other mediators regulated by IFN-γ, including those produced by classically-activated alveolar macrophages, IL-12, TNF-α and iNOS. In contrast, the level of expression of pro-inflammatory cytokines by lung cells slightly decreased at this time point and continued at a low level throughout subsequent observation periods. IFN-γ expression induced by the H27 strain led to potent macrophage activation inducing high levels of IL-12 and iNOS expression and was accompanied by a significant increase of IL-17. The increased levels of expression of the main mediators of anti-mycobacterial protection, IFN-γ, TNF-α and iNOS, detected in the lungs of mice infected with H27 bacteria by RT-PCR were confirmed by evaluation of secretion of corresponding gene products by lung cells obtained from the mice infected with studied mycobacterial strains. The lung cells, obtained on day 30 after infection with the H27 strain and cultured *in vitro* for 48 h, produced significantly higher levels of IFN-γ, TNF-α and NO than the cells infected with P104 strain (Fig. 7).

Following this, by day 60 post-infection, the level of IFN-γ and IL-12 expression induced by the H27 strain had reduced whereas the expression of iNOS and TNF-α remained stable (Fig. 6).

In the GKO mice, a lack of IFN-γ led to the increased expression of IL-17 and IL-4 lymphokines, although the kinetics of induction were different in the mice infected by the two studied *M. avium* strains. The H27 strain had already induced strong IL-17 expression by day 5 after infection (Fig. 6). The level of cytokine expression at this time point was six times higher than that induced in WT animals. In contrast to IL-17, enhanced induction of IL-4 by the H27 strain was observed 15 d after infection, which coincided with inhibition of IL-17 expression. Later on, the level of IL-4 started to progressively decrease, whereas expression of IL-17 increased and continued to be high up to day 60 of observation. The kinetics of IL-4 expression induced by the P104 strain was similar to that induced by the H27 strain, whereas induction of IL-17 was significantly less pronounced. It peaked 30 d after infection, but the level of induction was twice as low as that induced by the H27 bacteria and had dropped down further by day 60 post-infection (Fig. 5).

The lack of IFN-γ expression in GKO mice led to dramatic inhibition of the early induction of pro-inflammatory mediators IL-12 and iNOS, observed in the lungs of WT mice 5 d after infection. Expression of these molecules progressively increased reaching the peak at day 30, but the level of expression was significantly lower than that induced in WT mice by the H27 strain, which was able to induce strong mononuclear phagocyte activation mediated by IFN-γ. It should be noted that expression of all of the studied pro-inflammatory cytokines, including TNF-α which presented moderate and stable expression within 30 days, was significantly inhibited by day 60 post-infection with both *M. avium* strains.

## Discussion

In this study, we evaluated bacterial virulence and pathogenic properties of two epidemiologically unrelated *M. avium* clinical isolates, H27 and P104, which presented a similar genotype pattern, as determined by combined IS*1245*-based RFLP and PRA genotyping [12], suggesting a genetically-close relatedness of these strains. To explore the virulence, we examined the bacterial growth in murine bone marrow-derived macrophages and pathogenicity in a well-characterized model of pulmonary infection in mycobacteria-resistant WT C57BL/6 mice and mycobacteria-susceptible GKO C57BL/6 mice.

The intracellular growth of the H27 strain in the macrophage culture was significantly faster than of the P104 strain. In accordance with the *in vitro* data, the initial growth of H27 bacteria in the lungs, within 15 d after intratracheal infection of WT or GKO mice, was more pronounced as well. However, starting day 30 post-infection, gradual reduction of the bacterial burden in the lungs, as well as in livers and spleens, was observed, whereas the P104 strain was able to persist in these animals leading to chronic infection without any reduction in bacterial numbers. These data demonstrate that although an enhanced capacity to intracellular growth is a factor that could contribute to bacterial virulence, nevertheless, the *in vitro* growth characteristics of bacteria not always resembles those *in vivo*.

The suppression of growth of H27 bacteria coincided with an increase in IFN-γ expression in the lungs 30 days after infection. High level of pulmonary IFN-γ expression in C57BL/6 mice infected with *M. avium* reflected the establishment of the specific anti-mycobacterial Th1-type immune response 20 - 30 d after infection [17]. Our data confirmed that IFN-γ induction was essential for the control of the *M. avium* infection contributing to the development of the typical granulomatous response. In the mice deficient in IFN- γ the immune reaction was disorganized and characterized by diffuse granulomatous inflammation in the lungs. In these mice, both strains were able to grow in increasing CFU numbers in all of the tested organs. Reduction of the bacterial loads observed in the WT mice infected with H27 strain started 30 d post- infection, after induction of the strong IFN-γ expression, and was more pronounced at the 90 d time point. Although IFN-γ levels dropped at this time point, probably as a result of previously described phenomena of IFN-γ-dependent depletion of lymphocytes at a late stage of infection by pathogenic *M. avium* strains [18], nevertheless, the reduction of the bacterial numbers continued. Even the low levels of IFN-γ detected in the lungs of mice infected with moderately virulent bacteria were sufficient to maintain functional granulomas and to control the bacterial growth [17], [19].

The higher resistance of P104 bacteria observed in WT mice could be associated with a weaker immunostimulatory activity of this strain. This strain started to induce IFN-γ expression in the lungs of WT mice earlier than the H27 strain, but at a significantly lower level. Additionally, induction of IFN-γ in cells infected by the P104 strain was not accompanied by the enhancement of expression of mediators associated with classical macrophage activation, such as iNOS and IL-12, whereas in mice infected with the H27 strain, these mediators were induced by IFN-γ. The lower mRNA expression of IFN-γ, iNOS and TNF-α in the lungs infected with P104 bacteria was demonstrated to result in lower production of corresponding pro-inflammatory mediators by the lung cells, contributing to the persistence of P104 bacteria in mice. We can only speculate about the reason of lower immunostimulatory activity of P104 strain. Additional studies could elucidate whether it was associated with other known mechanisms of immune response alteration by *M. avium*, such as induction in the cells of SOCS1- and SOCS3- mediated pathways interfering with the IFN-γ responses [20], [21] or down-regulation of the IFN-γ receptor in the cells infected with some pathogenic *M. avium* strains [22]. It should be noted that neither strain examined in this study induced significant expression of inhibitory IL-10 cytokine. These data confirm the previous observations that C57BL/6 mice are low producers of IL-10 in response to infection by *M. avium* [23].

Although in GKO mice, the growth of both strains was significantly higher, it was slowed down after 60 d of infection. Relatively high level of IL-12 expression observed in these mice could contribute to induction of IL-17 expression that is known to be promoted synergistically by IL-12 and IL-23 cytokines generated by dendritic cells upon interaction with mycobacteria [24]. Recent data demonstrated the switch to Th17-type immunity in GKO mice infected with BCG [25], and our data suggest that this also takes place in GKO mice infected with *M. avium*. The role of the Th17-type immunity in mycobacterial infections was not well elucidated. In WT mice, IL-17 is an important factor contributing to IFN-γ-mediated granuloma formation and establishment of long-lasting protective immunity against mycobacteria [26], and in our experiments, highly immunostimulating H27 bacteria induced high levels of the IL-17 expression. In the absence of IFN-γ, which is required to control IL-17-producing T cells [25], enhanced expression of IL-17 could contribute to either protective mechanisms resulting in the inhibition of mycobacterial growth rate as observed in our experiments at the later stage of infection, or to the severe lung pathology typical for GKO mice infected with pathogenic mycobacteria, or to both of these effects.

The significant differences in the immunomodulating activity and growth capacity of the H27 and P104 isolates suggest that these bacteria have different phenotypic properties despite the fact that these isolates belong to the same cluster on the basis of combined RFLP-IS*1245* and PRA-*hsp65* typing. According to the standard view, mycobacterial isolates exhibiting indistinguishable fingerprinting patterns based on the analysis of the specific insertion elements (IS) can be considered direct progeny of the same bacterial clone, suggesting that they should have similar biological properties. This approach is currently used to trace transmission of *M. tuberculosis* strains and was suggested to be useful to type *M. avium* strains to the subspecies level according to the presence or absence of specific IS [8] while analysis of variations in IS copy number [27] and/or mycobacterial interspersed repetitive-unit-variable-number tandem-repeats, MIRU-VNTR [28], [29], was suggested to facilitate strain tracking. Nevertheless, recent data obtained as a result of massively parallel whole-genome sequencing of two *M. tuberculosis* isolates of Beijing genotype sharing the same DNA fingerprint demonstrated a high level of genomic diversity in these bacteria [30] suggesting that the observed differences could have impact on the biological properties of these closely related bacteria. In comparison with *M. tuberculosis,* genetic and phenotypic diversity of *M. avium* strains is much wider. Recent data obtained by employment of genomic sequence methodology demonstrated considerable heterogeneity among *M. avium* strains, especially *M. avium* subsp. *hominissuis* strains, including evidence of insertions/deletions, genetic exchange [31], and even homologous recombination as a result of horizontal gene transfer [32]. In this context, our data, providing evidence of differences in the virulence of *M. avium* subsp. *hominissuis* isolates presenting indistinguishable genotype patterns, could reflect sequence polymorphisms, most likely single nucleotide polymorphisms (SNPs), and/or homologous recombinations occurred in genomes of these genetically-closely related bacteria.

In contrast to direct airway patient-to-patient transmission of *M. tuberculosis*, transmission of *M. avium* is more complex and probably is mediated by environmental stages causing corresponding bacterial adaptations and increasing the opportunities for genetic exchange with other species or with other *M. avium* subsp. *hominissuis* organisms in the diverse environmental habitats of these bacteria. Considering the results obtained in this study, it is possible to speculate that the modulation of bacterial properties during growth in diverse environments can lead to differences in the immunomodulating properties and intracellular growth capacities of genotypically similar isolates.

In conclusion, the data obtained is this work demonstrated that genotype similarity determined by analysis of IS or single gene variations does not predict similarity in the virulence of *M. avium* isolates.

## Materials and Methods

### Ethics Statement

The mice were maintained in a Biosafety Level 3 animal vivarium in a specific pathogen-free facility at the Universidade Estadual do Norte Fluminense and were given sterile water, mouse chow, bedding enrichment for the duration of the experiments. All experiments were conducted under protocols reviewed and approved by the local Animal Care and Usage Committee.

### Mice and bacteria

Specific pathogen-free male and female C57BL/6 and IFN-γ^−/−^ (GKO) mice of the same genetic background, from 8 to 10 weeks old, were purchased from the Biotery of USP (São Paulo, Brazil) and CEMIB Biotery (UNICAMP, Campinas, Brazil), respectively. The GKO mice were identified genotypically by PCR of tail DNA.

*M. avium* isolates H27 (isolate from human blood with undetermined HIV infection status) and P104 (isolate from pig lymph node) were obtained in two distant regions of Brazil from two epidemiologically unrelated cases of *M. avium* infection [12]. These isolates showed indistinguishable genotype patterns belonging to cluster B determined by combined IS*1245*-based restriction fragment length polymorphism (RFLP) and PCR-restriction enzyme analysis (PRA) of *hsp65* [12]. On solid culture medium, the colonies of both bacterial strains displayed a smooth transparent colony phenotype typical for virulent *M. avium*. Bacteria were grown in complete Middlebrook 7H9 medium supplemented with 10% ADC (albumin, dextrose and catalase - Becton Dickinson, USA) and 0.05% Tween 80, at 37°C for 7 d until attaining mid-log phase. Aliquots of 1.0 ml containing 10^7^ to 10^8^ bacilli were frozen and stored at −70°C. Before use, bacterial aliquots were thawed, grown for 3 d in complete 7H9 medium and then suspended in sterile PBS to obtain the desired concentrations. The bacterial capacity to grow in 7H9 broth was measured by spectrophotometry. Bacterial suspensions adjusted to OD_600nm_ of 0.066 were cultured at 37°C for 24 days with daily agitation. Bacterial tubes were then vortexed, ultrasonicated in a water bath, and the optical density of suspension was measured. To confirm the lack of significant alteration in OD_600nm_ readings, colony forming units (CFU) were determined for each culture on day 0 through the plating of appropriate bacterial dilution onto 7H10 agar supplemented with OADC (oleic acid, albumin, dextrose and catalase - Becton Dickinson, USA).

### Cell culture of bone marrow-derived macrophages and macrophage infection

Bone marrow-derived macrophages (BMDM) were generated from the femurs of C57BL/6 mice as described previously [33]. BMDM cells were infected by P104 and H27 *M. avium* strains at a multiplicity of infection (MOI) 1∶1 bacterium/macrophage. After 3 h incubation, the cultures were washed 3 times with 1x PBS and cultured in DMEM-F12 medium containing 2% fetal bovine serum (FBS) for 6 d at 37°C. The bacterial uptake was evaluated 3 h after infection by quantification of the percentage of macrophages with internalized mycobacteria in the cultures stained by Ziehl-Neelsen method. Macrophage viability was monitored daily by trypan-blue staining of the cells in the well and evaluation of the cell viability and monolayer integrity under inverted microscopy. The cell viability in infected and control cultures was similar throughout the experiment (approx. 95%). For quantification of the intracellular mycobacteria the CFU test was employed. The cultures were lysed by 0.1% saponin. Serially diluted and viable bacteria were tested by plating onto 7H10 agar supplemented with OADC. Colonies were counted after 10 d of incubation at 37°C.

### Experimental infections in mice

Mice were anesthetized by intraperitoneal injection with 250 µl of 2.5% 2,2,2-tribromoetanol (Acrós Organics, New Jersey, USA). The trachea was exposed via a small midline incision, and *M. avium* bacterial suspensions (60 µl, 2.5×10^6^ bacilli/mouse) were inoculated intratracheally (i.t.). The incision was sutured with sterile silk. At days 0, 5, 15, 30, 60 and 90 following infection**,** bacterial loads in the lungs were analyzed. Bacterial loads in the spleens and livers, cytokine mRNA expression in the lungs, and histopathological alterations in the lungs, liver and spleen, were analyzed at time points indicated in the text. Bacterial counts were determined by plating serial dilutions of homogenates of lungs (right lung), livers and spleens on nutrient 7H10 agar supplemented with 10% OADC and counting CFUs after two weeks incubation at 37°C. Bacterial counts were referred to as the log_10_ CFU per organ. For each of the mice strains, a total of six animals were infected for each time point.

### Histopathological analysis in mice

The left lung, the spleen and the liver from each mouse were fixed in 10% neutral aqueous buffered formalin and subsequently embedded in paraffin. Four micrometer-thick sections were stained with hematoxylin-eosin (H&E) to analyze tissue alterations and by the Ziehl-Neelsen method to detect the presence of alcohol-acid resistant bacilli (BAAR). The samples were examined under a Olympus BX-41 microscope (Tokyo, Japan) and the images were captured by Coolpix P995 (Nikon)- coupled device camera.

### Real-time PCR analysis of cytokine mRNA expression in lung

Total cellular RNA was isolated using Trizol Reagent (Invitrogen, NY, USA) from homogenate of right lungs. Total RNA was extracted using acid phenol-guanidinium thiocyanate-cloroform extraction method, according to the manufacturer's recommendations. Quality and quantity of RNA before and after the samples treatment with DNAse (Fermentas, Germany) were evaluated through spectrophotometry (260 nm/280 nm) and on agarose gels [34].

Two micrograms of each RNA sample were used to generate cDNA by High-Capacity cDNA Reverse Transcription Kits (Applied Biosystems, USA) according to the manufacturer's instructions. Quantitative real-time PCR was performed in a Lightcyler 1.0 (Roche Diagnostics, Germany) using a Lightcycler FastStart DNA Master SYBR Green I kit (Roche Diagnostics). For PCR amplification, 360 ηg cDNA was used. The primer pairs were designed using the Primer Express (Applied Biosystems) program for the targets presented in Table 1. Melting curve analysis of the amplified products was used to check the quality of the PCR products. Each result was normalized by the housekeeping β-actin gene expression. Quantification of gene expression was performed using the Pfaffl method [35].

**Table 1:** Nucleotide sequences of primers used in the RT-PCR.

	Primer sequence (5′-3′)	
Cytokine	Forward	Reverse
**IL-12p35**	CCACTGGAACTACACAAGAACG	GCACAGGGTCATCATCAAAG
**IL-17**	AACATGAGTCCAGGGAGAGC	GCTGAGCTTTGAGGGATGAT
**TNF-α**	ATGGCCTCCCTCTCATCAGT	CACTTGGTGGTTTGCTACGA
**iNOS**	TCAAGTGGCATAGATGTGGAAG	TGGGTCCTCTGGTCAAACTC
**IL-10**	TCAGCCAGGTGAAGACTTTCT	TCATTTCCGATAAGGCTTGG
**IL-4**	GCAACGAAGAACACCACAGA	CTGCAGCTCCATGAGAACAC
**IFN-γ**	TCAAGTGGCATAGATGTGGAAG	TGGCTCTGCAGGATTTTCAT
**β-actin** a	GACGGCCAGGTCATCACTAT	ATGCCACAGGATTCCATACC

a Housekeeping reporter gene.

### Cell culture of lung cells from infected mice and evaluation of nitric oxide and cytokine production *ex vivo*

Single-cell suspensions were prepared from the lungs obtained on day 5, 15 and 30 after infection. The dissected lung tissue was incubated with RPMI-1640 medium containing collagenase type II (2 µg/ml; Gibco) and type IV bovine pancreatic DNase (1 µg/ml; Roche Diagnostics) for 45 min at 37°C. The digested lungs were further disrupted by pushing the tissue through a cell strainer. Red blood cells were lysed with lysing buffer, containing (0.144 M NH_4_Cl, 0.0169 M TRIS base, pH 7.4), washed, and resuspended in complete RPMI medium supplemented with 10% FBS. The obtained cell numbers were determined using a hemocytometer. The cells were cultured in 96 well-plates, 5×10^4^ cells/well, at 37°C. After 48 h of incubation, culture supernatants were harvested, filter sterilized to eliminate mycobacteria, and stored at −80°C. After thawing the supernatants, concentrations of IFN-γ and TNF-α were measured by sandwich ELISA employing OptEIA kits (BD Biosciences) according to manufacturer's instructions. Nitric oxide production was measured by Griess reaction. Briefly, 50 µl supernatant was incubated with 50 µl Griess reagent for 5 min at room temperature, and the NO_2_-concentration was determined by measuring the optical density (OD) at 550 nm in reference to a standard NaNO_2_ solution. Assays were completed with duplicate samples and results are expressed as the mean of two experiments.

### Statistical analysis

Statistical significance was determined using the two-way ANOVA test with Bonferroni post-hoc test, employing Prism 4 software (GraphPad, San Diego, CA). A p-value of<0.05 was considered to indicate a significant difference.

## References

[1] Porter JD (1996). Mycobacteriosis and HIV infection: the new public health challenge.. J Antimicrob Chemother.

[2] Remus N, Reichenbach J, Picard C, Rietschel C, Wood P (2001). Impaired interferon gamma-mediated immunity and susceptibility to mycobacterial infection in childhood.. Pediatr Res.

[3] Prince DS, Peterson DD, Steiner RM, Gottlieb JE, Scott R (1989). Infection with *Mycobacterium avium* complex in patients without predisposing conditions.. N Engl J Med.

[4] Dhillon SS, Watanakunakorn C (2000). Lady Windermere syndrome: middle lobe bronchiectasis and *Mycobacterium avium* complex infection due to voluntary cough suppression.. Clin Infect Dis.

[5] Field SK, Cowie RL (2006). Lung disease due to the more common nontuberculous mycobacteria.. Chest.

[6] Benson C (1994). Disseminated *Mycobacterium avium* complex disease in patients with AIDS.. AIDS Res Hum Retroviruses.

[7] Turenne CY, Wallace R, Behr MA (2007). *Mycobacterium avium* in the postgenomic era.. Clin Microbiol Rev.

[8] Ritacco V, Kremer K, van der Laan T, Pijnenburg JE, de Haas PE (1998). Use of IS*901* and IS*1245* in RFLP typing of *Mycobacterium avium* complex: relatedness among serovar reference strains, human and animal isolates.. Int J Tuberc Lung Dis.

[9] van Soolingen D, Bauer J, Ritacco V, Leao SC, Pavlik I (1998). IS*1245* restriction fragment length polymorphism typing of *Mycobacterium avium* isolates: proposal for standardization.. J Clin Microbiol.

[10] Mijs W, de Haas P, Rossau R, Van der Laan T, Rigouts L (2002). Molecular evidence to support a proposal to reserve the designation ‘*Mycobacterium avium* subsp. *Avium*’ for bird-type isolates and ‘*M. avium* subsp. *Hominissuis*’ for the human/porcine type of *M. avium*.. Int J Syst Evol Microbiol.

[11] Horan KL, Freeman R, Weigel K, Semret M, Pfaller S (2006). Isolation of the genome sequence strain *Mycobacterium avium* 104 from multiple patients over a 17-year period.. J Clin Microbiol.

[12] Oliveira RS, Sircili MP, Oliveira EMD, Balian SC, Ferreira-Neto JS (2003). Identification of *Mycobacterium avium* genotypes with distinctive traits by combination of IS*1245*-based Restriction Fragment Length Polymorphism and Restriction Analysis of hsp65.. J Clin Microb.

[13] Aly S, Laskay T, Mages J, Malzan A, Lang R (2007). Interferon-gamma-dependent mechanisms of mycobacteria-induced pulmonary immunopathology: the role of angiostasis and CXCR3-targeted chemokines for granuloma necrosis.. J Pathol.

[14] Tateishi Y, Hirayama Y, Ozeki Y, Nishiuchi Y, Yoshimura M (2009). Virulence of *Mycobacterium avium* complex strains isolated from immunocompetent patients.. Microb Pathog.

[15] Ordway D, Henao-Tamayo M, Shanley C, Smith EE, Palanisamy G (2008). Influence of *Mycobacterium bovis* BCG vaccination on cellular immune response of guinea pigs challenged with *Mycobacterium tuberculosis*.. Clin Vaccine Immunol.

[16] Pearl JE, Saunders B, Ehlers S, Orme IM, Cooper AM (2001). Inflammation and lymphocyte activation during mycobacterial infection in the interferon-gamma-deficient mouse.. Cell Immunol.

[17] Florido M, Goncalves AS, Silva RA, Ehlers S, Cooper AM (1999). Resistance of virulent *Mycobacterium avium* to gamma interferon-mediated antimicrobial activity suggests additional signals for induction of mycobacteriostasis.. Infect Immun.

[18] Florido M, Pearl JE, Solache A, Borges M, Haynes L (2005). Gamma interferon-induced T-cell loss in virulent *Mycobacterium avium* infection.. Infect Immun.

[19] Florido M, Goncalves AS, Gomes MS, Appelberg R (2004). CD40 is required for the optimal induction of protective immunity to *Mycobacterium avium*.. Immunology.

[20] Vazquez N, Greenwell-Wild T, Rekka S, Orenstein JM, Wahl SM (2006). *Mycobacterium avium*-induced SOCS contributes to resistance to IFN-gamma-mediated mycobactericidal activity in human macrophages.. J Leukoc Biol.

[21] Blumenthal A, Lauber J, Hoffmann R, Ernst M, Keller C (2005). Common and unique gene expression signatures of human macrophages in response to four strains of *Mycobacterium avium* that differ in their growth and persistence characteristics.. Infect Immun.

[22] Hussain S, Zwilling BS, Lafuse WP (1999). *Mycobacterium avium* infection of mouse macrophages inhibits IFN-gamma Janus kinase-STAT signaling and gene induction by down-regulation of the IFN-gamma receptor.. J Immunol.

[23] Florido M, Appelberg R (2006). Genetic control of immune-mediated necrosis of *Mycobacterium avium* granulomas.. Immunology.

[24] Khader SA, Cooper AM (2008). IL-23 and IL-17 in tuberculosis.. Cytokine.

[25] Cruz A, Khader SA, Torrado E, Fraga A, Pearl JE (2006). Cutting edge: IFN-gamma regulates the induction and expansion of IL-17-producing CD4 T cells during mycobacterial infection.. J Immunol.

[26] Curtis MM, Way SS (2009). Interleukin-17 in host defence against bacterial, mycobacterial and fungal pathogens.. Immunology.

[27] Bartos M, Hlozek P, Svastova P, Dvorska L, Bull T (2006). Identification of members of *Mycobacterium avium* species by Accu-Probes, serotyping, and single IS*900*, IS*901*, IS*1245* and IS*901*-flanking region PCR with internal standards.. J Microbiol Methods.

[28] Radomski N, Thibault VC, Karoui C, de Cruz K, Cochard T (2010). Determination of genotypic diversity of *Mycobacterium avium* subspecies from human and animal origins by mycobacterial interspersed repetitive-unit-variable-number tandem-repeat and IS*1311* restriction fragment length polymorphism typing methods.. J Clin Microbiol.

[29] Tirkkonen T, Pakarinen J, Rintala E, Ali-Vehmas T, Marttila H (2010). Comparison of variable-number tandem-repeat markers typing and IS*1245* restriction fragment length polymorphism fingerprinting of *Mycobacterium avium* subsp. *hominissuis* from human and porcine origins.. Acta Vet Scand.

[30] Niemann S, Koser CU, Gagneux S, Plinke C, Homolka S (2009). Genomic diversity among drug sensitive and multidrug resistant isolates of *Mycobacterium tuberculosis* with identical DNA fingerprints.. PLoS One.

[31] Turenne CY, Collins DM, Alexander DC, Behr MA (2008). *Mycobacterium avium* subsp. *paratuberculosis* and *M. avium* subsp. *avium* are independently evolved pathogenic clones of a much broader group of *M. avium* organisms.. J Bacteriol.

[32] Krzywinska E, Krzywinski J, Schorey JS (2004). Naturally occurring horizontal gene transfer and homologous recombination in *Mycobacterium*.. Microbiology.

[33] Racoosin EL, Swanson JA (1989). Macrophage colony-stimulating factor (rM-CSF) stimulates pinocytosis in bone marrow-derived macrophages.. J Exp Med.

[34] Sambrook J, Russell DW (2001). Molecular cloning: a laboratory manual.3rd.ed.. :Cold Spring Harbor Laboratory.

[35] Pffaffl MW (2001). A new mathematical model for relative quantification for real time PCR.. Nucleic Acids Research.

